# Quantitative performance and optimal regularization parameter in block sequential regularized expectation maximization reconstructions in clinical ^68^Ga-PSMA PET/MR

**DOI:** 10.1186/s13550-018-0414-4

**Published:** 2018-07-27

**Authors:** Edwin E. G. W. ter Voert, Urs J. Muehlematter, Gaspar Delso, Daniele A. Pizzuto, Julian Müller, Hannes W. Nagel, Irene A. Burger

**Affiliations:** 1 Department of Nuclear Medicine, University Hospital Zurich, Rämistrasse 100, CH-8091 Zurich, Switzerland; 2 University of Zurich, Rämistrasse 71, CH-8006 Zurich, Switzerland; 3 GE Healthcare, 3000 N Grandview Blvd, Waukesha, WI 53188 USA; 4 Institute of Nuclear Medicine, Università Cattolica del Sacro Cuore, Largo Francesco Vito 1, 00168 Rome, Italy

**Keywords:** BSREM, Q.Clear, Regularization parameter, ^68^Ga-PSMA, Pelvic area, Clinical research, PET/MR

## Abstract

**Background:**

In contrast to ordered subset expectation maximization (OSEM), block sequential regularized expectation maximization (BSREM) positron emission tomography (PET) reconstruction algorithms can run until full convergence while controlling image quality and noise. Recent studies with BSREM and ^18^F-FDG PET reported higher signal-to-noise ratios and higher standardized uptake values (SUV). In this study, we investigate the optimal regularization parameter (*β*) for clinical ^68^Ga-PSMA PET/MR reconstructions in the pelvic region applying time-of-flight (TOF) BSREM in comparison to TOF OSEM.

Two-minute emission data from the pelvic region of 25 patients who underwent ^68^Ga-PSMA PET/MR were retrospectively reconstructed. Reference OSEM reconstructions had 28 subsets and 2 iterations. BSREM reconstructions were performed with 15 *β* values between 150 and 1200. Regions of interest (ROIs) were drawn around lesions and in uniform background. Background SUVmean (average) and SUVstd (standard deviation), and lesion SUVmax (average of 5 hottest voxels) were calculated. Differences were analyzed using the Wilcoxon matched pairs signed-rank test.

**Results:**

A total of 40 lesions were identified in the pelvic region. Background noise (SUVstd) and lesions SUVmax decreased with increasing *β*. Image reconstructions with *β* values lower than 400 have higher (*p* < 0.01) background noise, compared to the reference OSEM reconstructions, and are therefore less useful. Lesions with low activity on images reconstructed with *β* values higher than 600 have a lower (*p* < 0.05) SUVmax compared to the reference. These reconstructions are likely visually appealing due to the lower background noise, but the lower SUVmax could possibly render small low-uptake lesions invisible.

**Conclusions:**

In our study, we showed that PET images reconstructed with TOF BSREM in combination with the ^68^Ga-PSMA tracer result in lower background noise and higher SUVmax values in lesions compared to TOF OSEM. Our study indicates that a *β* value between 400 and 550 might be the optimal compromise between high SUVmax and low background noise.

**Electronic supplementary material:**

The online version of this article (10.1186/s13550-018-0414-4) contains supplementary material, which is available to authorized users.

## Background

Image reconstruction in positron emission tomography (PET) is the process of forming an image data set that represents the spatial distribution of activity in the patient by using the detected coincidence events. The basic algorithm used since the mid-1970s to reconstruct the PET images is filtered back-projection (FBP) [1]. Along with developments in computing power, new maximum likelihood (ML)-based reconstruction methods were developed that included accurate statistical Poisson-based noise models and physical modeling [2]. These ML-based models were later combined with expectation–maximization (EM) algorithms [3]. Compared to FBP, iterative reconstructions led, in most situations, to an improvement in (streaking) artifacts, noise, and resolution as it allowed accurate noise and physics/system models to be included [4–7]. Although the ML-EM reconstructions are accurate, the total reconstruction time is long due to the substantial computing power required per iteration and the many iterations required before convergence is reached. To accelerate the reconstruction process, new methods like ordered subset expectation maximization (OSEM) were developed [8]. In OSEM, the measured data is divided into subsets (or blocks) and the EM algorithm is applied to each of these subsets [9]. When all subsets are processed, the next iteration starts. The OSEM method is fast and currently one of the most applied in PET reconstructions. OSEM is, in contrast to ML-EM, not a true ML estimator; it does not converge to a maximum likelihood image [10]. Moreover, since image noise increases with iterations, ML-EM and OSEM algorithms are usually stopped before the image becomes unacceptably noisy. Typically, some post-filtering is applied to enhance the images [11].

Several studies reported quantitative differences comparing FBP to OSEM reconstructions [12, 13]. Most of these could be explained by other effects then the reconstruction process, like differences in attenuation correction, reconstruction filters, higher noise levels in FBP, or ROI selection [14]. Higher uptake values are sometimes reported for OSEM, but this is almost completely reversible by equalizing the image resolution [5, 15].

In most cases, OSEM provides accurate quantitative results within 3%; however, larger biases (up to 50%) can be expected in regions with a 5- to 10-fold hotter background [5]. This can partially be explained by differences in convergence rate in different regions. Cold regions within a hotter background converge at a different rate than hot regions within a colder background [5]. Stopping early with iterating could therefore result in non-uniform recovery of activity [16]. This means that, although the resulting images are visually appealing, e.g., hotspot detection in oncology, there could be inaccuracies in a quantitative assessment as the reconstruction algorithm did not reach full “convergence” in all image parts. Moreover, considering the high activity in the urinary bladder and kidneys, and the low background activity and higher tumor to background ratios with ^68^Ga-PSMA, the quantitative performance of OSEM may be reduced.

In addition to the previously mentioned reconstruction methods, there are also regularized iterative reconstruction methods. The penalized likelihood image reconstruction methods, like block sequential regularized expectation maximization (BSREM), add to the likelihood function a penalty function that controls image quality [9, 17–20]. Due to this penalty function, which provides activity-dependent noise control and edge preservation while iterating, BSREM can run until full convergence is reached [21]. This means that all image parts are fully converged, thereby increasing the accuracy in a quantitative assessment. Until recently, the penalized likelihood image reconstruction methods were not commonly used. Apart from the longer reconstruction times compared to OSEM, the resulting images of the edge-preserving penalized likelihood methods showed patchy background textures and other undesirable features [22]. However, new developments show promising results.

In a study by Asma et al. [23], lesions were inserted into multiple clinical whole-body PET/CT datasets in representative locations. These ‘hybrid’ datasets combine clinically realistic image backgrounds with known lesion activity. They found superior quantitation over early stopped and post-filtered OSEM, while maintaining clinically acceptable image quality. Ahn et al. [21] extended this study with more clinical datasets from multiple clinical sites and included phantom measurements. Their results also demonstrated improvements in lesion quantitation accuracy compared to OSEM, especially in cold background regions such as lungs. Teoh et al. [24] performed a phantom and clinical study. They found that the BSREM reconstructions were preferred over OSEM. A clinical evaluation study by Sah et al. [25] indicated that time-of-flight (TOF) BSREM reconstructions showed the best results in all categories, independent of body compartments, compared to TOF OSEM. Due to recent improvements and these promising results, the BSREM algorithms are also becoming commercially available (e.g., Q.Clear, GE Healthcare, Waukesha, WI, USA).

Although the abovementioned studies show promising results with 2-deoxy-2-[^18^F]-fluoro-D-glucose (^18^F-FDG) on PET, the situation could be different for the ^68^Gallium-labeled tracer targeting the prostate-specific membrane antigen (^68^Ga-PSMA) as it has a clearly different uptake pattern compared to the ^18^F-FDG tracer used in most studies. The low background activity, higher tumor to background ratios, higher positron energy, and larger positron range could, e.g., have an effect on the performance [20, 26]. Moreover, previous studies were performed on PET/computed tomography (CT) whereas in this study, a PET/magnetic resonance (MR) is applied. This could also lead to differences as the current clinical PET/MR scanners have no attenuation coefficients for the bone in the MR-based attenuation map, except for the skull.

As BSREM runs until full convergence, the number of iterations and subsets are no controlling parameters in BSREM. BSREM does, however, have a regularization parameter *β*. It controls the global strength of the regularization, the relative difference penalty function.

Therefore, in this study, we investigate the optimal regularization parameter *β* for clinical ^68^Ga-PSMA PET/MR reconstructions in the pelvic region applying TOF BSREM in comparison to TOF OSEM.

## Methods

### Patients

From a total of 125 patients referred to ^68^Ga-PSMA PET/MRI between June 2016 and January 2017, we retrospectively included 25 patients (median age 71 years, range 42–79; median body mass index 25.4 kg/m^2^, range 19.6–36.0 kg/m^2^) with ^68^Ga-PSMA PET positive pelvic lesions that provided written informed consent for retrospective use of their data. Of these, 16 patients were scanned for biochemical recurrence after radical prostatectomy and 9 patients were scanned for staging of a newly diagnosed high-risk prostate cancer. The study has been approved by the cantonal ethics committee.

### PET/MR imaging

To reduce the kidney, ureters, and urinary bladder activity during the scan, furosemide was injected intravenously (0.13 mg/kg) 30 min prior to ^68^Ga-PSMA-11 injection and patients were asked to void prior to the scan [27]. After a standardized uptake time of 60 min, a clinical routine whole-body TOF PET/MRI (SIGNA PET/MR, GE Healthcare, Waukesha, WI, USA) was performed. For a similar scanner, the average spatial resolution in full with at half maximum (FWHM) for ^18^F at 1 cm off center was reported to be 4.2 mm, and 5.1 mm at 10 cm off center [28]. The spatial resolution (FWHM) for ^68^Ga was 5.46, 5.26, and 6.10 mm (*x*, *y*, and *z*, respectively) in air and 5.63, 4.77, and 6.47 mm (*x*, *y*, and *z*, respectively) in water [29]. The per crystal TOF timing resolution was less than 400 ps [28, 30].

Patients were positioned in supine position with the arms down. Clinical whole-body scans consist of 6 bed positions (2 min per bed), from the vertex of the skull to the mid-thighs. A 15-min 1 bed position PET/MR examination of the pelvic area was performed prior to or after the whole-body scan. Only 2-min emission data of the 15-min emission data was applied in this study to have datasets that are comparable to the clinical scan [31]. During PET scanning, a default MR acquisition for attenuation correction was performed. For the pelvic area, additional anatomical MR sequences were acquired including an axial *T*_1_-weighted fast spin echo and an axial and coronal *T*_2_-weighted fast recovery fast spin echo. Dynamic contrast-enhanced sequences and diffusion-weighted imaging were added when deemed necessary for clinical evaluation.

### PET reconstructions

All PET reconstructions (3D-TOF-OSEM and 3D-TOF-BSREM) were performed on a workstation running a PET reconstruction toolbox (PETtoolbox R1.28 MP24, GE Healthcare, Waukesha, WI, USA) for MATLAB (MATLAB R2017a, The MathWorks Inc., Natick, MA, USA). All PET reconstructions included the standard corrections like decay, scatter, random, dead time, attenuation, normalization, and the detector response. The reconstruction diameter was 60 cm, and the image grid was 256 × 256 with 2.34 × 2.34 × 2.78 mm^3^ voxels. All OSEM reconstructions were post-filtered in image space using an in-plane Gaussian convolution kernel with a full-width-at-half-maximum of 5.0 mm, followed by a standard axial filter with a three-slice kernel using relative weights of 1:4:1. These settings are commonly used in centers with a similar PET/MR scanner and result in optimal clinical images [27, 31]. BSREM reconstructions do not use post-filtering.

The PETtoolbox uses PET sinogram data and MR-based attenuation maps for the PET reconstructions. These datasets were copied from the PET/MR scanner to the workstation. All PET sinograms were created on the PET/MR scanner using 2-min TOF emission data from the pelvic region. All MR-based attenuation maps were created on the PET/MR scanner using a continuous fat-water-based attenuation correction method [32].

#### BSREM reconstructions and the *β* value

The penalized likelihood function of BSREM is mathematically expressed as:

1 $$ \widehat{x}=\arg {\max}_{x\ge 0}{\sum}_i^{n_d}{y}_i\log \left({\left[ Px\right]}_i+{b}_i\right)-\left({\left[ Px\right]}_i+{b}_i\right)-\beta R(x) $$

where *n*_*d*_ denotes the total number of detector pairs; *y*_*i*_represents the measured PET coincidence data in the sinogram; *x* is the activity image estimate; *P* is the forward projection operator including attenuation, normalization, and point spread function resolution modeling; *b* denotes the estimated background contributions of randoms and scatter; *R(x)* is the regularization or penalty function to control noise and edge preservation; and *β* controls the global strength of the regularization [21, 23].

The relative difference penalty, which has the advantage of providing activity-dependent noise control, was introduced in [33, 34] and is given by

2 $$ R(x)={\sum}_{j=1}^{n_v}{\sum}_{k\in {N}_j}{w}_j{w}_k\frac{{\left({x}_j-{x}_k\right)}^2}{\left({x}_j+{x}_k\right)+\gamma \left|{x}_j-{x}_k\right|} $$

where *n*_*v*_ denotes the number of voxels, *N*_*j*_ denotes the set of neighbors of voxel *j*, *w*_*j*_ and *w*_*k*_ are the position-dependent weights controlling the local smoothing level at voxel *j*, and *γ = 2* which controls edge preservation [21, 23].

Unless specified otherwise, TOF BSREM reconstructions were initialized by 2 iterations of non-TOF OSEM and 3 iterations of non-TOF BSREM followed by 8 iterations of TOF BSREM, all with 28 subsets.

#### Reference 3D TOF OSEM reconstructions

As true SUV values are not known in clinical data, we used the 3D TOF OSEM reconstructions with 2 iterations and 28 subsets as reference for all other reconstructions. The applied 3D TOF OSEM reconstruction settings are the same as the clinical settings and were chosen as these resulted in the optimal PET images for the specified indication [31]. In addition, 3D TOF OSEM reconstructions with 3 iterations and 28 subsets were performed, as a reference for too high background noise.

#### The effect of the number of iterations/subsets

To demonstrate the effect of the number of iterations and subsets on the image noise, we performed 2 series of reconstructions in one patient with 5 lesions: The first series was performed with 3D TOF OSEM using 28 subsets and 1, 2, 3, 4, 6, 8, 10, 12, 14, 16, 20, and 25 iterations. The second series was performed with 3D TOF BSREM using *β* values of 350, 400, 450, 500, and 700. With the PETtoolbox, we can set the number of subsets and the maximum number of iterations. This allowed us to stop the iterating process after 1, 2, 3, 4, 6, 8, 10, 12, 14, 16, 20, and 25 iterations with 28 subsets for each previously listed *β* value.

#### The effect of the regularization parameter

After demonstrating the effect of the number of iterations and subsets, we studied the effect of reconstruction parameter *β*, controlling the global strength of the regularization. For this, 3D TOF BSREM reconstructions were performed for all patient datasets using a range of 15 *β* values 150, 200, 250, 300, 350, 400, 450, 500, 550, 600, 700, 800, 900, 1000, and 1200. The lesions were divided into 3 sets according to their size and uptake, as measured on the reference 3D TOF OSEM reconstructions: A set of lesions having a small size (≤ 1 cm^3^) and low uptake (SUVmax ≤ 5 g/ml), a set of lesions having a large size (> 10 cm^3^) or a high uptake (SUVmax > 10 g/ml), and a set with the remaining lesions having medium size (1 cm^3^ < volume ≤ 10 cm^3^) and medium uptake (5 g/ml < SUVmax ≤ 10 g/ml).

### Image analysis

All images were analyzed on dedicated workstations (Advantage Workstation 4.6, GE Healthcare) and PMOD (v3.8, PMOD-Technologies LLC, Zurich, Switzerland), which allowed images to be viewed side-by-side as well as in fused mode. Regions of interest (ROIs) around lesions were drawn manually by an experienced nuclear medicine/radiology physician using PET, MR, and fused images on the PMOD workstation. Lesion size was automatically calculated based on the ROIs. For the background measurements, ROIs were drawn in uniform uptake regions in adipose/muscle tissue lateral and posterior to the hip joint. Next, the average standardized uptake value (SUVmean) and the standard deviation (SUVstd) in the background ROIs were calculated. The SUVstd was considered a measure for the background noise, and the uniform region allowed the measurement of small differences in noise [35]. In every lesion, the SUVmax was defined and calculated by averaging the SUV values of the 5 hottest voxels to reduce the statistical noise induced by a single hottest voxel [36].

In this study, we also defined a contrast recovery (CR) ratio comparing the tumor to background ratio of a TOF BSREM reconstruction (numerator) to the tumor to background ratio of the reference TOF OSEM reconstruction (denominator):

3 $$ CR=\frac{\left(\mathrm{SUVmax}/\mathrm{SUVmean}\_\mathrm{bkgnd}\right)-1}{\left(\mathrm{SUVmax}\_\mathrm{ref}/\mathrm{SUVmean}\_\mathrm{bkgnd}\_\mathrm{ref}\right)-1} $$

where SUVmax is obtained from the lesion, SUVmean from the background (‘bkgnd’), and ‘ref’ indicates the reference 3D TOF OSEM reconstructions with 2 iterations and 28 subsets.

### Statistical analyses

Statistical analyses were performed using Prism 7 (GraphPad Software Inc., San Diego, California, USA). Differences between medians were ascertained and compared using the Wilcoxon matched-pairs signed-rank test as it could not be proven (D’Agostino-Pearson omnibus normality test) that our distributions were Gaussian (except for the high (> 900) *β* value reconstructions that are blurred most). A difference was considered to be statistically significant when *p* < 0.05.

## Results

The median-injected tracer dose was 128 MBq (range 105–161 MBq) which means a whole-body effective dose of 3.0 mSv. The average time difference between tracer injection and pelvis scan time was 53 min (standard deviation 21 min). After the PET images were reconstructed, a visual inspection was performed to exclude, e.g., reconstruction failures. All images were found to be as expected for the applied settings.

### The effect of the number of iterations/subsets

**Fig. 1:**
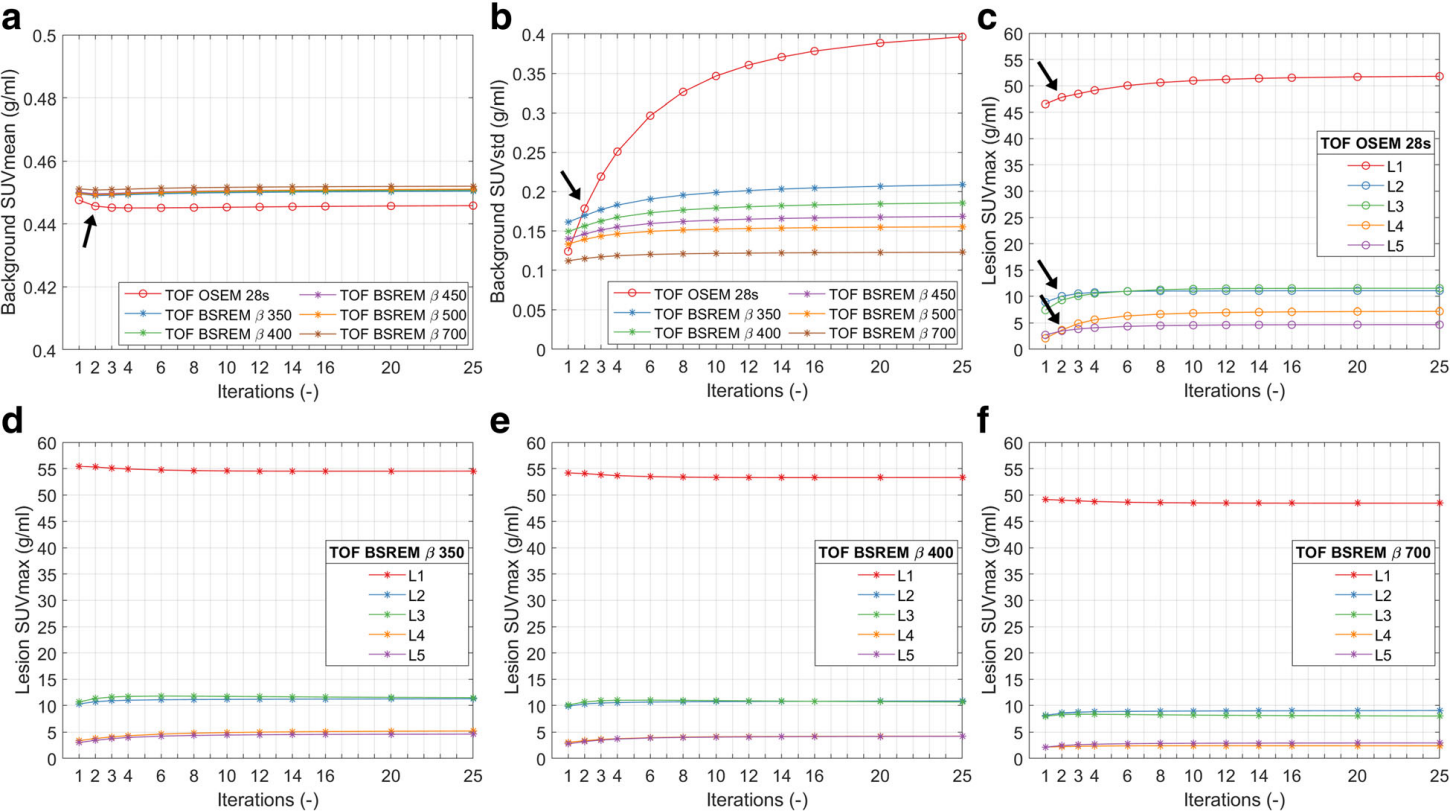
Example dataset showing the effect of increasing iterations. The first two panels show the background SUVmean (**a**) and the SUVstd (**b**), as a function of the number of iterations, for TOF OSEM with 28 subsets and for TOF BSREM with regularization parameter *β* = 350, 400, 450, 500, and 700. The remaining 4 panels show the lesion SUVmax as a function of the number of iterations for 5 lesions (L1–L5) for TOF OSEM with 28 subsets (**c**) and for TOF BSREM with regularization parameter *β* = 350 (**d**), 400 (**e**), and 700 (**f**). The arrows indicate the default reference TOF OSEM reconstruction with 28 subsets and 2 iterations. SUV standardized uptake value, SUVmean the average value of the voxels in the background ROI, SUVstd the standard deviation of the values of the voxels in the background ROI, SUVmax the average of the hottest 5 voxels in the lesion, TOF time-of-flight, OSEM ordered subset expectation maximization, BSREM block sequential regularized expectation maximization

**Fig. 2:**
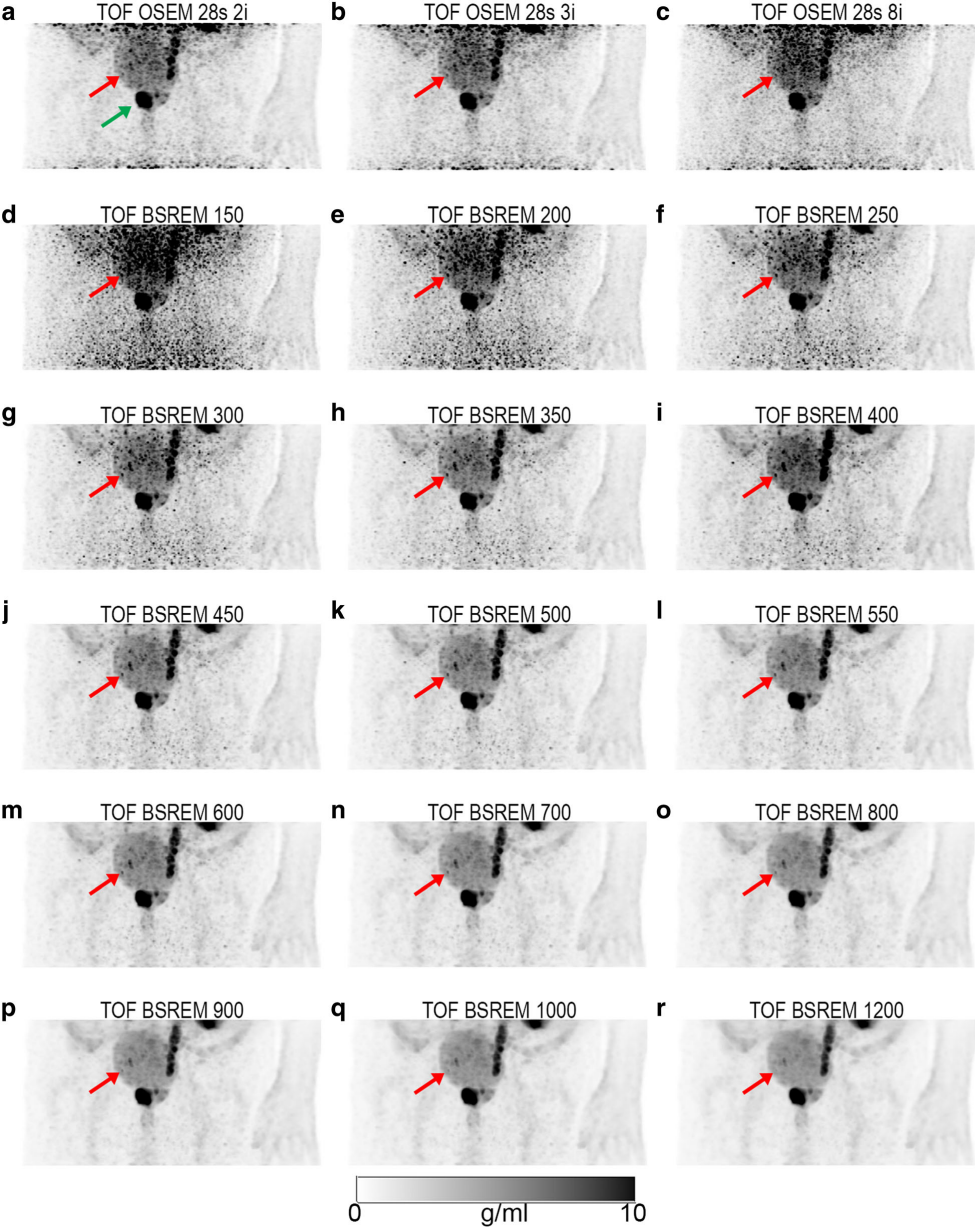
Example dataset showing coronal PET maximum intensity projections (MIP) of the pelvic frame. PSMA accumulation is seen in the left ureter; besides the high activity in the primary tumor (green arrow), a small lymph node can be detected (red arrow). The reconstructions are TOF OSEM with 28 subsets and 2 (**a**), 3 (**b**), and 8 (**c**) iterations; TOF BSREM with regularization parameter *β* = 150 (**d**), 200 (**e**), 250 (**f**), 300 (**g**), 350 (**h**), 400 (**i**), 450 (**j**), 500 (**k**), 550 (**l**), 600 (**m**), 700 (**n**), 800 (**o**), 900 (**p**), 1000 (**q**), and 1200 (**r**). All images have SUV scale 0–10 g/ml. SUV standardized uptake value, TOF time-of-flight, OSEM ordered subset expectation maximization, BSREM block sequential regularized expectation maximization

The effect of the number of iterations/subsets on the reconstructed PET images is demonstrated in Fig. 1. We see that the number of iterations has a limited effect on SUVmean for both TOF OSEM and TOF BSREM reconstructions (Fig. 1a). However, we also see that the image noise (background SUVstd) increases significantly with the number of iterations in TOF OSEM (Fig. 1b). Too high noise levels can hamper clinical evaluations; and therefore, these TOF OSEM reconstructions are stopped after 2 iterations (Fig. 1b, at the arrow). This can also be visually appreciated in Fig. 2a–c where we see an increase of image noise in the TOF OSEM reconstructions with 2, 3, and 8 iterations, respectively.

In contrast to the TOF OSEM reconstructions, the image noise in TOF BSREM is (after the first few iterations) relatively constant (almost independent of the number of iterations) and its level depends on the *β* value (Fig. 1b). The TOF BSREM reconstructions with a *β* value of 400 and higher appear to have an equal or even lower background noise than the TOF OSEM with 2 iterations (Fig. 1b, at the arrow).

The example patient dataset included 5 lesions (L1–5 in Fig. 1c–f) with volumes of 5.9, 0.3, 1.2, 0.4, and 0.3 cm^3^, respectively. The lesions were ordered according to their SUVmax in the reference TOF OSEM reconstruction with 28 subsets and 2 iterations (Fig. 1c, at the arrows). With only 2 iterations, it is likely that not all image parts are fully converged. If we would increase the number of iterations from 2 to 25, the lesion SUVmax would be closer to the real SUVmax of a fully converged image. We can see (Fig. 1c) that the SUVmax of the lesions is different at 2 iterations and 25 iterations. While with OSEM, we have to limit the number of iterations to 2 (due to the increase in image noise), with BSREM, we could iterate until full convergence. With TOF BSREM, the *β* value not only controls the noise (Fig. 1b), it also affects SUVmax (Fig. 1d–f). The higher the *β* value, the lower the SUVmax and the lower the image noise.

### The effect of the regularization parameter

**Fig. 3:**
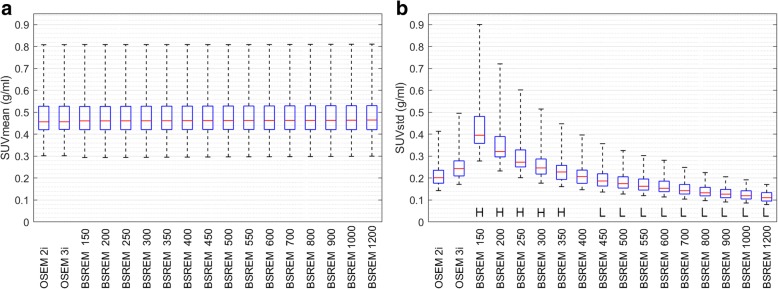
Box and whisker plots showing SUVmean and SUVstd distributions from background ROIs in 25 patients. The left panel shows SUVmean (**a**) and the right panel shows SUVstd (**b**). Each panel shows from left to right the results of the TOF OSEM reconstructions with 28 subsets and 2 and 3 iterations, followed by TOF BSREM reconstructions with regularization parameter *β* = 150, 200, 250, 300, 350, 400, 450, 500, 550, 600, 700, 800, 900, 1000, and 1200. *H* indicates significantly higher values, and *L* indicates significantly lower parameter values for TOF BSREM compared to reference TOF OSEM. TOF time-of-flight, SUV standardized uptake value, SUVmean the average value of the voxels in the background ROI, SUVstd the standard deviation of the values of the voxels in the background ROI, OSEM ordered subset expectation maximization, BSREM block sequential regularized expectation maximization

A total of 40 lesions were identified in the pelvic area of 25 patients. The box and whisker plots in Fig. 3 show similar background SUVmean values for both TOF OSEM reconstructions with 2 and 3 iterations, and all 15 TOF BSREM reconstructions with varying *β* values (see also Table 1). The image noise (SUVstd), however, decreases with increasing *β* values. A *β* value of 300 has approximately the same (too high) noise values (0.26 ± 0.07 g/ml, mean ± standard deviation) as the TOF OSEM reconstruction with 3 iterations and 28 subsets (0.26 ± 0.07 g/ml). A *β* value of 400 has, on average, the same image noise (0.21 ± 0.05 g/ml) as the reference TOF OSEM reconstruction with 28 subsets and 2 iterations (0.22 ± 0.06 g/ml) (see also Table 1). A *β* value higher than 400 results, on average, in lower image noise as the reference TOF OSEM reconstruction. The ‘H’ in Fig. 3b indicates TOF BSREM reconstructions with significantly higher background noise compared to the reference TOF OSEM with 28 subsets and 2 iterations. These reconstructions are not recommended for clinical evaluations. This can also be visually appreciated in Fig. 2d–r where we see a decrease of image noise in the TOF BSREM reconstructions going from *β* = 150 to *β* = 1200.

**Table 1:** The percentage increase/decrease for different parameters comparing 8 TOF BSREM reconstructions with reference TOF OSEM reconstructions

		TOF BSREM							
		*β* = 300	*β* = 350	*β* = 400	*β* = 450	*β* = 500	*β* = 550	*β* = 600	*β* = 700
Background SUVmean		0.0% (0.60)	0.0% (0.63)	0.0% (0.65)	0.1% (0.69)	0.1% (0.67)	0.1% (0.69)	0.1% (0.75)	0.1% (0.87)
Background SUVstd		22.0% (< 0.01) *H*	8.3% (< 0.01) *H*	−1.0% (0.41)	− 9.2% (< 0.01) *L*	− 15.2% (< 0.01) *L*	−18.4% (< 0.01) *L*	− 21.0% (< 0.01) *L*	−27.0% (< 0.01) *L*
Small size, low-uptake lesions (*N* = 8)	SUVmax	28.0% (0.02) *H*	17.7% (0.15)	8.0% (0.55)	−1.6% (0.74)	− 8.1% (0.25)	−12.8% (0.11)	− 18.1% (0.05) *L*	− 24.6% (0.02) *L*
	CR	32.4% (0.02) *H*	20.2% (0.15)	8.1% (0.55)	− 2.8% (0.74)	−10.0% (0.15)	−15.4% (0.08)	− 21.6% (0.05) *L*	− 29.3% (0.02) *L*
Medium size, medium uptake lesions (*N* = 15)	SUVmax	27.0% (< 0.01) *H*	18.6% (< 0.01) *H*	14.5% (0.01) *H*	10.5% (0.08)	− 0.4% (0.64)	− 3.3% (0.42)	− 5.8% (0.21)	− 10.6% (< 0.01) *L*
	CR	28.2% (< 0.01) *H*	20.4% (< 0.01) *H*	15.6% (0.02) *H*	8.8% (0.15)	0.1% (0.89)	− 2.9% (0.42)	− 5.5% (0.06)	−12.4% (< 0.01) *L*
Large size, high-uptake lesions (*N* = 17)	SUVmax	16.3% (< 0.01) *H*	11.5% (< 0.01) *H*	7.0% (< 0.01) *H*	4.1% (0.01) *H*	1.8% (0.19)	0.3% (0.43)	− 1.7% (0.89)	− 4.9% (0.03) *L*
	CR	17.4% (< 0.01) *H*	12.5% (< 0.01) *H*	9.1% (< 0.01) *H*	6.2% (< 0.01) *H*	2.9% (0.08)	0.2% (0.46)	− 1.9% (0.71)	− 5.2% (0.02) *L*

The regularization parameter applied in TOF BSREM is indicated by the *β* value (top). *P* values are listed in parentheses. *H* indicates significantly higher values, and *L* indicates significantly lower parameter values for TOF BSREM compared to reference TOF OSEM with 28 subsets and 2 iterations *SUV* standardized uptake value, *SUVmax* the average of the hottest 5 voxels in the lesion, *CR* the percentage increase of the tumor to background ratio of the indicated reconstruction compared to the reference TOF OSEM reconstruction with 2 iterations and 28 subsets, *TOF* time-of-flight, *OSEM* ordered subset expectation maximization, *BSREM* block sequential regularized expectation maximization

**Fig. 4:**
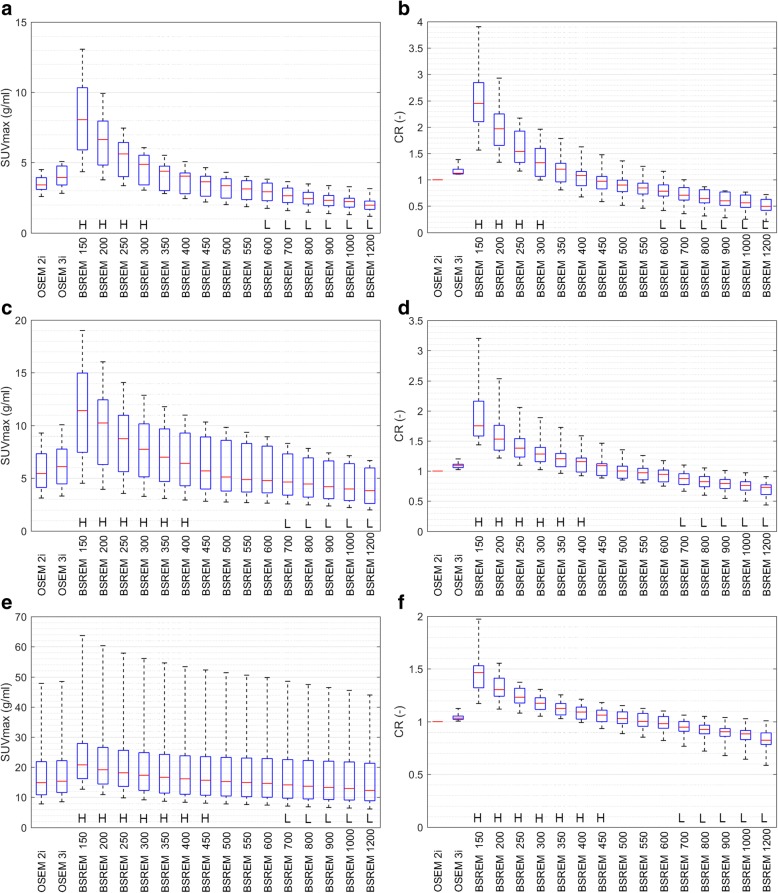
Box and whiskers plots showing SUVmax and CR distributions obtained from lesions in 25 patients. The left side shows SUVmax (**a**, **c**, and **e**), and the right side CR (**b**, **d**, and **f**). The top row (**a**, **b**) shows the group with small size and low-uptake lesions (*N* = 8), the middle row (**c**, **d**) shows the group with medium size and medium uptake lesions (*N* = 15), and the bottom row (**e**, **f**) shows the group with large size or high-uptake lesions (*N* = 17). Each subplot shows from left to right the results of the TOF OSEM reconstructions with 28 subsets and 2 and 3 iterations, followed by TOF BSREM reconstructions with regularization parameter *β* = 150, 200, 250, 300, 350, 400, 450, 500, 550, 600, 700, 800, 900, 1000, 1200. *H* indicates significantly higher values and *L* indicates significantly lower parameter values for TOF BSREM compared to reference TOF OSEM. CR = the tumor to background ratio of the indicated TOF reconstruction compared to the reference TOF OSEM reconstruction with 2 iterations and 28 subsets (Eq. 3), a CR value higher than 1 indicates a better tumor to background ratio for BSREM compared to OSEM. TOF time-of-flight, SUV standardized uptake value, SUVmax the average of the hottest 5 voxels in the lesion, OSEM ordered subset expectation maximization, BSREM block sequential regularized expectation maximization

The effect of *β* on lesion SUVmax and CR is shown in Fig. 4 for the 3 lesion subgroups: small size, low uptake; medium size, medium uptake; and large size, high uptake. A low *β* value of up to 350 will result in higher SUVmax values compared to the reference TOF OSEM, but also in higher image noise (Fig. 3 and Table 1). These reconstructed images are not clinically useful as the noise renders the lesions indistinguishable from background noise. A *β* value between 400 and 550 will, on average, result in higher or approximately equal SUVmax compared to the reference, with nearly equal or lower background noise (Fig. 4 and Table 1). A *β* value higher than 600 will result in not only lower SUVmax values but also lower background noise. The “L” in Fig. 4 indicates TOF BSREM reconstructions with significantly lower SUVmax compared to the reference TOF OSEM with 28 subsets and 2 iterations. These BSREM reconstructions are not recommended for clinical evaluations where accurate SUV values are required. Due to the lower background noise, tumor detection could be easier, but this has to be proven in future studies.

**Fig. 5:**
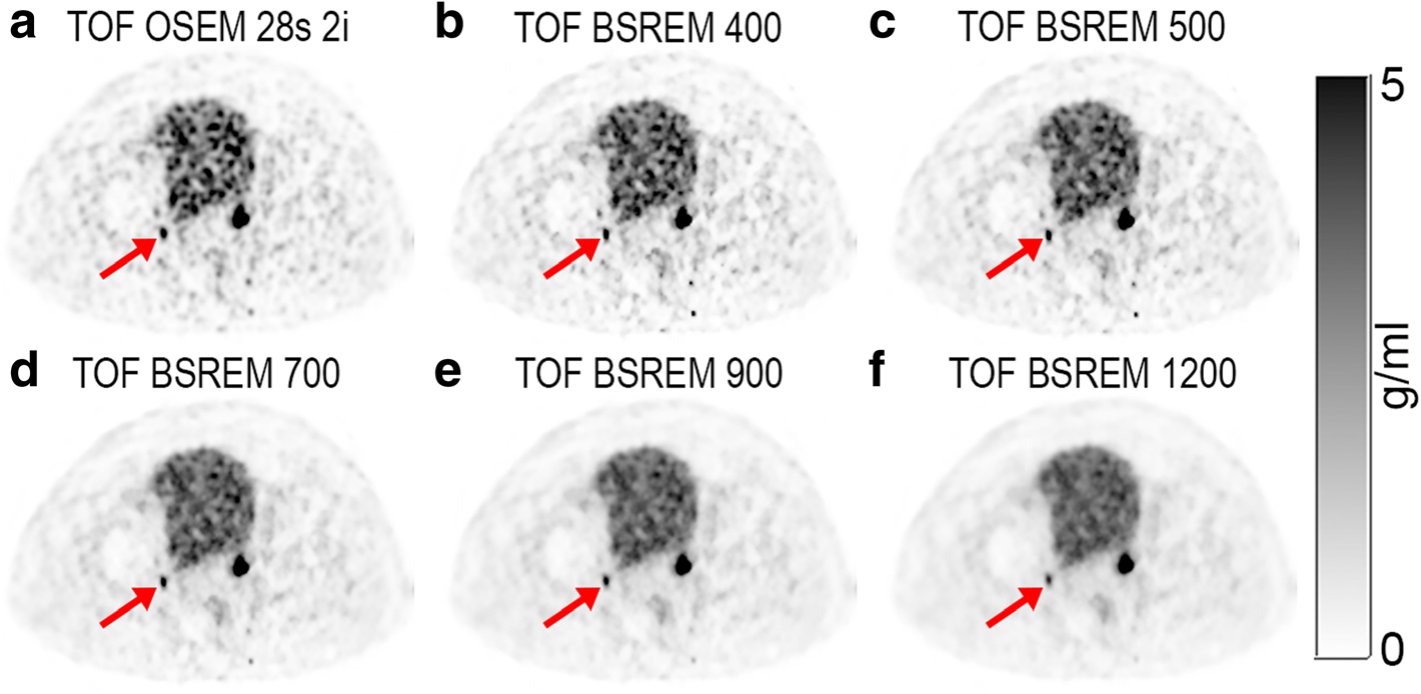
Example dataset showing axial PET images of the pelvic frame. The reconstructions are TOF OSEM with 28 subsets and 2 iterations (**a**); TOF BSREM with regularization parameter *β* = 400 (**b**), 500 (**c**), 700 (**d**), 900 (**e**), and 1200 (**f**). All images have SUV scale 0–5 g/ml. Red arrow indicates the same lymph node lesion as in Fig. 2. It can be appreciated that the background noise decreases with increasing *β* values. It can also be appreciated that the SUV of the lymph node lesion decreases with increasing *β* values; however, the lesion is still clearly detectable even with *β* = 1200 (**f**). SUV standardized uptake value, TOF time-of-flight, OSEM ordered subset expectation maximization, BSREM block sequential regularized expectation maximization

This can also be visually appreciated in Fig. 2 showing a large lesions with high uptake in the prostate (green arrow) and a small lymph node lesion with medium uptake (red arrow). Going from *β* = 400 to *β* = 1200 (Fig. 2i–r), we see that the SUV of the small lymph node lesion decreases. On the higher *β* value images, the lymph node lesion has a similar SUV as the urinary bladder, making it disappear on the coronal maximum intensity projection (MIP) images. Figure 5 shows the same lymph node lesion on axial images. We see that the lesion is located at a small distance from the urinary bladder. Due to this distance and due to the decrease in background noise with higher *β* values, it is still possible to detect the lymph node lesion on the *β* = 1200 image (Fig. 5f). Compared to the reference TOF OSEM (SUVmax = 7.3 g/ml), the SUVmax is higher on the TOF BSREM *β* = 400 (9.6 g/ml), but lower on the TOF BSREM *β* = 1200 (4.5 g/ml).

**Fig. 6:**
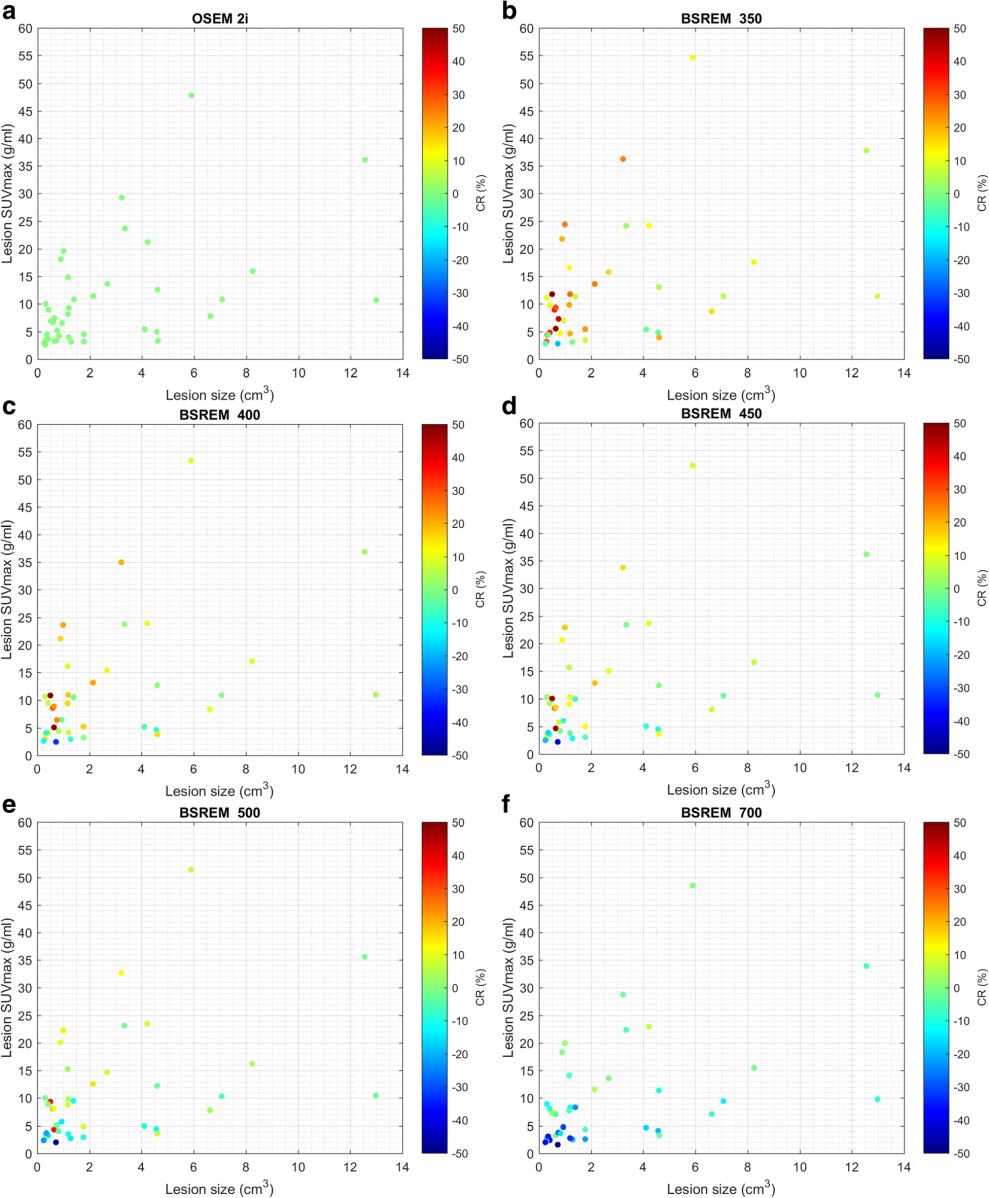
Scatterplots showing lesion size, SUVmax, and CR increase obtained in 25 patients. Each panel represents a different reconstruction: TOF OSEM with 28 subsets and 2 iterations (**a**), TOF BSREM with regularization parameter *β* = 350 (**b**), 400 (**c**), 450 (**d**), 500 (**e**), and 700 (**f**). The CR range was limited from − 50 to 50% for clarity. It can be appreciated that with increasing *β* value, the SUVmax and CR decreases in all lesions. SUV standardized uptake value, SUVmax the average of the hottest 5 voxels in the lesion, CR the percentage increase of the tumor to background ratio of the indicated reconstruction compared to the reference TOF OSEM reconstruction with 2 iterations and 28 subsets. TOF time-of-flight, OSEM ordered subset expectation maximization, BSREM block sequential regularized expectation maximization

Figure 6 shows for each lesion its size, SUVmax, and the percentage change in CR compared to the reference TOF OSEM with 28 subsets and 2 iterations, which is also shown in Fig. 6a. With increasing *β*, the SUVmax and CR decrease for most lesions. Although all lesions are affected, the percentage decrease is larger for small lesions with low uptake. The lesion with the highest SUVmax, for example, has an approximate SUVmax value of 48 g/ml in TOF OSEM with 28 subsets and 2 iterations, and 55, 53, 52, 51, and 49 g/ml in TOF BSREM *β* = 350, 400, 450, 500, and 700, respectively. The corresponding CR is 13, 11, 8, 6, and 3% higher than BSREM compared to OSEM. With TOF BSREM *β* = 400, most lesions have higher SUVmax, although some low-uptake lesions have lower SUVmax compared to TOF OSEM and although most lesions have increased CR, several low-uptake lesions have decreased CR. With TOF BSREM *β* = 700 most lesions have lower SUVmax, and most low-uptake lesions have decreased CR, while high-uptake lesions have similar CR. This is in agreement with the results in Fig. 4 and Table 1, but now, the individual lesion with a specific SUVmax and size is shown.

## Discussion

We investigated the effect of the regularization parameter *β* in TOF BSREM reconstructions of clinical ^68^Ga-PSMA PET acquisitions in the pelvic area. Our quantitative results indicate that the best *β* value for pelvic ^68^Ga-PSMA PET with TOF BSREM reconstructions is in the range between 400 and 550 for 2-min emission data and a median-injected dose of 128 MBq.

A *β* value near 400 will result in images having a similar background noise level and higher SUVmax values compared to reference TOF OSEM with 2 iterations and 28 subsets. These images could be used for clinical evaluations where accurate SUV values are required, like treatment evaluations and follow-up cases. A *β* value near 550 will result in images having a lower background noise level and similar SUVmax values. The low background noise could make tumor detection easier, but this has to be proven in future studies. Lower *β* values will result in higher SUVmax and more background noise, and higher *β* values will result in lower SUVmax and less background noise. The lower the *β* value, the lower the effect of the regularization function and the more the penalized likelihood function behaves like a normal likelihood function without noise control.

One clinical study performed with ^18^F-FDG on PET/CT found optimal *β* values in the range 350–400 for an injected mean dose of 297 MBq, 60 min uptake time, and 2 min per bed scans [25]. A phantom/clinical study with ^18^F-FDG on PET/CT, with an injected dose of 288 MBq, 90 min uptake time, and 4 min per bed scans, found an optimal *β* value of 400 [24]. These findings are slightly below the findings in our study. A higher injected dose and/or longer scan time could be a possible explanation for the lower *β* values in their study. Both ^18^F-FDG studies also noted that higher *β* values resulted in lower SUVmax.

Despite the fact that the penalized likelihood algorithms already exist for many years [17, 19], and the fact that the relative difference penalty by Nuyts et al. [33, 34], the BSREM by De Pierro et al. [37], and the combination by Ahn et al. [10], were already introduced some 15 years ago, these type of reconstruction methods were not used commonly in clinical routine. With todays improved computing power and after recent quantitative evaluations by, e.g., Asma et al. [23, 38] and Ahn et al. [21] and clinical evaluations by, e.g., Ma et al. [39], Passalaqua et al. [40], Teoh et al. [24], and Sah et al. [25], which showed promising results, the TOF BSREM reconstruction method is now attracting more attention and is considered a possibly better alternative to OSEM.

Although OSEM reconstructions are fast, the early termination of the iteration process required to limit the noise, results in different convergence rates in different image regions. The TOF BSREM-penalized likelihood method applied in this study on the other hand achieves effectively full convergence, thereby improving the quantitative accuracy [21]. The more accurate higher SUVmax values can play a crucial role in detecting small lesions, especially in regions with high background uptake like the liver or brain in ^18^F-FDG PET. Due to the slow convergence rates of OSEM in cold regions, like the lungs in ^18^F-FDG, or in regions near hot spots, like the kidneys or urinary bladder, BSREM’s full convergence results in more accurate measurements in these areas [21, 24]. Besides the improved quantitative accuracy, the increase in SUVmax and the lower background noise with BSREM could possibly also be applied to further reduce the injected tracer dose, but this has to be proven in future studies.

Simulated phantom measurements are an optimal way to investigate certain aspects under specific circumstances, as parameters, like the ground truth, are known. Previous studies included “hybrid” phantom measurements in which lesions with a known activity were inserted in real patient datasets at interesting locations, near hotspots and in cold backgrounds [21]. In this study, we used clinical patient data which has the advantage of a diverse patient population with lesions in commonly occurring locations. The disadvantage is that the ground truth is not available. We, however, used the same datasets for OSEM and BSREM, meaning that all parameters are the same, except for the reconstruction methods. This means that differences were only due to the differences in reconstruction methods.

The definition of SUVmax, as well as the ROI size and resolution can have a significant impact on measurements [14, 41]. In our study, we defined SUVmax as the average of the 5 hottest voxels, as the variability of this metric was found to be the lowest, compared to several other measures like the hottest voxel in a lesion [36, 42]. As all datasets in our study were obtained with the same scanner, and used for both the OSEM and BSREM reconstructions, it should not affect our results. Care has to be taken when comparing our results with other scanners or definitions.

In this study, for each dataset, the variable *β* was systematically increased in small steps and the resulting BRSEM reconstructions were compared with one OSEM reconstruction. The probability values (in Table 1) were, however, not corrected for multiple comparisons. As adding more tests by “subsampling” the *β* value range will normally not result in accidental positive findings, we believe that it is better not to apply a correction.

This study has some limitations. The current study was, for example, performed on a relatively new TOF PET/MR system. Although we would expect comparable results on similar PET/MR or PET/CT systems, results may vary.

Significant halo artifacts have been reported to occur on many PET/CT and PET/MR systems with the use of 68Ga-PSMA-11. This is mainly due to high organ-to-background activity ratios between the bladder/kidneys and surrounding soft tissue, and due to incorrect scatter correction algorithms [43, 44]. In those cases, the administration of furosemide is recommended to substantially reduce bladder activity [27]. The scanners in our institution have a second generation scatter correction algorithm installed, and, as a result, halo artifacts are rarely seen [45, 46]. Our patient cohort was reconstructed with the latest versions, and no halo artifacts were visible (Additional file 1: Figure S1 in Pizzuto et al. [47] shows comparable scans).

Tissues surrounding high-activity areas, like the urinary bladder, could suffer from SUV overestimation as activity could spill into this region from the high-activity area [48]. Proposed solutions range from bladder voiding by urinary catheterization to new segmentation and reconstruction methods [49]. In our study, the administration of furosemide lowered the urinary bladder activity to a SUVmean of 7.7 g/ml (range 2.5–20.9 g/ml, interquartile range 8.9 g/ml, decay corrected). Three cases with the highest urinary bladder SUV showed similar surrounding SUV values as 3 cases with the lowest urinary bladder uptake. Therefore, the spill-in effect was considered minimal in our study.

Although we included a wide range of patients with different BMI values, the presented results are also expected to be related to the acquisition conditions (dose, uptake time, acquisition time, resolution, etc.) as specified in the materials section.

The pelvis region contains a large percentage of the bone, and MR-based attenuation correction (MR-AC) does generally not incorporate bone attenuation (except for the head). As a result, the average SUVmean of normal tissue in the pelvis region was found to have a bias of − 18.7% for non-TOF and − 10.8% for TOF, and lesions near the bone were reported to have a bias of − 5.2% for non-TOF and − 4.6% for TOF, compared to PET/CT using ^18^F-FDG and ^18^F-choline [50]. Leynes et al. [51] performed zero echo time (ZTE) scans in the pelvis region, which allows the imaging of the bones with MR. They segmented the data and combined it with the normal Dixon-based MR-AC map to include bone attenuation in their ^18^F-FGD TOF OSEM reconstructions. For bone lesions in the pelvis region, they found a SUVmax bias of − 10.8% comparing normal MR-AC to CT-AC, which was reduced to − 3.17% when applying their new hybrid ZTE method. For soft tissue lesions in the pelvis region, they found a SUVmax bias of − 7.67% which was further reduced to − 3.54%. In our study, the MR-based AC maps were the same for OSEM and BSREM and had no bone attenuation incorporated. When bone tissue would be included in the AC map, it would improve both the OSEM as well as the BSREM results. We did not investigate the effect of the bone in the attenuation map in the comparison between OSEM and BSREM, but we expect that BSREM will be superior to OSEM, considering the results obtained in other studies applying ^18^F-FDG PET/CT, which include the bone [21]. It would be an interesting subject for future studies.

In this study, we did not perform a visual evaluation of the clinical image quality or the clinical significance. Therefore, it is unknown if TOF BSREM would lead to different diagnoses in ^68^Ga-PSMA PET of the pelvic area. However, the complex effects of the different *β* values on the behavior of absolute values, as well as on contrast and background, warranted a solid preliminary analysis of a wide range of *β* values. The current results give more insight and can be used to limit the amount of reconstructed PET images and thus evaluations to only those that are the most promising for further clinical investigations, which need to be performed in larger cohorts.

## Conclusions

As ^18^F-FDG is the most commonly applied PET tracer, most studies evaluating the quantitative properties on PET images reconstructed with (TOF) BSREM were performed with this tracer. In our study, we showed that PET images reconstructed with TOF BSREM in combination with the ^68^Ga-PSMA tracer also results in lower background noise and higher SUVmax values in lesions, compared to TOF OSEM. Our study indicates that a *β* value between 400 and 550 might be an optimal compromise between high SUVmax and low background noise. Larger studies need to be performed to assess the clinical benefit of BSREM over OSEM.

## Additional file

Additional file 1: **Figure S1.** Box and whisker plots showing tumor-to-background ratio distributions obtained from lesions in 25 patients. The top panel (a) shows the group with small size and low-uptake lesions (*N* = 8), the middle panel (b) shows the group with medium size and medium uptake lesions (*N* = 15), the lower panel (c) shows the group with large size or high-uptake lesions (*N* = 17). Each subplot shows from left to right the results of the TOF OSEM reconstructions with 28 subsets and 2 and 3 iterations, followed by TOF BSREM reconstructions with regularization parameter *β* = 150, 200, 250, 300, 350, 400, 450, 500, 550, 600, 700, 800, 900, 1000, and 1200. The tumor-to-background is defined as the SUVmax of the lesion devided by the SUVmean of the background. TOF = time-of-flight, SUV = standardized uptake value, SUVmax = the average of the hottest 5 voxels in the lesion, SUVmean = the average value of the voxels in the background ROI, OSEM = ordered subset expectation maximization, and BSREM = block sequential regularized expectation maximization. (DOCX 174 kb)

## Abbreviations

^18^F-FDG: 2-Deoxy-2-[^18^F]-fluoro-D-glucose
3D: Three dimensional
^68^Ga-PSMA: ^68^Gallium-labeled tracer targeting the prostate-specific membrane antigen
AC: Attenuation correction
BKG: Background
BSREM: Block sequential regularized expectation maximization
CR: Contrast recovery
CT: Computed tomography
EM: Expectation–maximization
FBP: Filtered back-projection
ML: Maximum likelihood
MR: Magnetic resonance
OSEM: Ordered subset expectation maximization
PET: Positron emission tomography
REF: Reference
ROI: Region of interest
SUV: Standardized uptake value
TOF: Time-of-flight

## References

[1] Radon J (1986). On the determination of functions from their integral values along certain manifolds. IEEE Trans Med Imaging.

[2] Rockmore AJ, Macovski A (1976). A maximum likelihood approach to emission image reconstruction from projections. IEEE Trans Nucl Sci.

[3] Shepp LA, Vardi Y (1982). Maximum likelihood reconstruction for emission tomography. IEEE Trans Med Imaging.

[4] Shepp LA, Vardi Y, Ra JB, Hilal SK, Cho ZH (1984). Maximum likelihood PET with real data. IEEE Trans Nucl Sci.

[5] Boellaard R, van Lingen A, Lammertsma AA (2001). Experimental and clinical evaluation of iterative reconstruction (OSEM) in dynamic PET: quantitative characteristics and effects on kinetic modeling. J Nucl Med.

[6] Johnson CA, Seidel J, Carson RE, Gandler WR, Sofer A, Green MV (1997). Evaluation of 3D reconstruction algorithms for a small animal PET camera. IEEE Trans Nucl Sci.

[7] Tsoumpas C, Turkheimer FE, Thielemans K (2008). Study of direct and indirect parametric estimation methods of linear models in dynamic positron emission tomography. Med Phys.

[8] Hudson HM, Larkin RS (1994). Accelerated image reconstruction using ordered subsets of projection data. IEEE Trans Med Imaging.

[9] Qi J, Leahy RM (2006). Iterative reconstruction techniques in emission computed tomography. Phys Med Biol.

[10] Ahn S, Fessler JA (2003). Globally convergent image reconstruction for emission tomography using relaxed ordered subsets algorithms. IEEE Trans Med Imaging.

[11] Liow JS, Strother SC (1991). Practical tradeoffs between noise, quantitation, and number of iterations for maximum likelihood-based reconstructions. IEEE Trans Med Imaging.

[12] Etchebehere EC, Macapinlac HA, Gonen M, Humm K, Yeung HW, Akhurst T (2002). Qualitative and quantitative comparison between images obtained with filtered back projection and iterative reconstruction in prostate cancer lesions of (18)F-FDG PET. Q J Nucl Med.

[13] Lonneux M, Borbath I, Bol A, Coppens A, Sibomana M, Bausart R (1999). Attenuation correction in whole-body FDG oncological studies: the role of statistical reconstruction. Eur J Nucl Med.

[14] Krak NC, Boellaard R, Hoekstra OS, Twisk JW, Hoekstra CJ, Lammertsma AA (2005). Effects of ROI definition and reconstruction method on quantitative outcome and applicability in a response monitoring trial. Eur J Nucl Med Mol Imaging.

[15] Vriens D, Visser EP, de Geus-Oei LF, Oyen WJ (2010). Methodological considerations in quantification of oncological FDG PET studies. Eur J Nucl Med Mol Imaging.

[16] Liow JS, Strother SC (1993). The convergence of object dependent resolution in maximum likelihood based tomographic image reconstruction. Phys Med Biol.

[17] Geman S, Geman D (1984). Stochastic relaxation, Gibbs distributions, and the Bayesian restoration of images. IEEE Trans Pattern Anal Mach Intell.

[18] Geman S, McClure D (1985). Bayesian image analysis methods: an application to single photon emission computed tomography.

[19] Mumcuoglu EU, Leahy RM, Cherry SR (1996). Bayesian reconstruction of PET images: methodology and performance analysis. Phys Med Biol.

[20] Asma E, Manjeshwar R (2007). Analysis of organ uniformity in low count density penalized likelihood PET images.

[21] Ahn S, Ross SG, Asma E, Miao J, Jin X, Cheng L (2015). Quantitative comparison of OSEM and penalized likelihood image reconstruction using relative difference penalties for clinical PET. Phys Med Biol.

[22] Chlewicki W, Hermansen F, Hansen SB (2004). Noise reduction and convergence of Bayesian algorithms with blobs based on the Huber function and median root prior. Phys Med Biol.

[23] Asma E, Ahn S, Ross SG, Chen A, Manjeshwar RM (2012). Accurate and consistent lesion quantitation with clinically acceptable penalized likelihood images.

[24] Teoh EJ, McGowan DR, Macpherson RE, Bradley KM, Gleeson FV (2015). Phantom and clinical evaluation of the Bayesian penalized likelihood reconstruction algorithm Q.Clear on an LYSO PET/CT system. J Nucl Med.

[25] Sah BR, Stolzmann P, Delso G, Wollenweber SD, Hullner M, Hakami YA (2017). Clinical evaluation of a block sequential regularized expectation maximization reconstruction algorithm in 18F-FDG PET/CT studies. Nucl Med Commun.

[26] Bertolli O, Eleftheriou A, Cecchetti M, Camarlinghi N, Belcari N, Tsoumpas C (2016). PET iterative reconstruction incorporating an efficient positron range correction method. Phys Med.

[27] Fendler WP, Eiber M, Beheshti M, Bomanji J, Ceci F, Cho S (2017). (68)Ga-PSMA PET/CT: joint EANM and SNMMI procedure guideline for prostate cancer imaging: version 1.0. Eur J Nucl Med Mol Imaging.

[28] Boellaard R, Quick HH (2015). Current image acquisition options in PET/MR. Semin Nucl Med.

[29] Huang S-y, Savic D, Yang J, Shrestha U, Seo Y. The effect of magnetic field on positron range and spatial resolution in an integrated whole-body time-of-flight PET/MRI system. IEEE Nucl Sci Symp Conf Rec. 2014;2014 10.1109/NSSMIC.2014.7431006. 10.1109/NSSMIC.2014.7431006 PMC4828037 27076778

[30] Levin C, Glover G, Deller T, McDaniel D, Peterson W, Maramraju SH (2013). Prototype time-of-flight PET ring integrated with a 3T MRI system for simultaneous whole-body PET/MR imaging. J Nucl Med Meeting Abstracts.

[31] Gandhi H, Holley D, Gulaka P, Iagaru A (2017). 68Ga-PSMA 11 PET/MRI influence of acquisition time on image quality. J Nucl Med.

[32] Wollenweber SD, Ambwani S, Lonn AHR, Shanbhag DD, Thiruvenkadam S, Kaushik S (2013). Comparison of 4-class and continuous fat/water methods for whole-body, MR-based PET attenuation correction. IEEE Trans Nucl Sci.

[33] Nuyts J, Beque D, Dupont P, Mortelmans L (2002). A concave prior penalizing relative differences for maximum-a-posteriori reconstruction in emission tomography. IEEE Trans Nucl Sci.

[34] Nuyts J, Michel C, Brepoels L, De Ceuninck L, Deroose C, Goffin K (2009). Performance of MAP reconstruction for hot lesion detection in whole-body PET/CT: an evaluation with human and numerical observers. IEEE Trans Med Imaging.

[35] Karp JS, Surti S, Daube-Witherspoon ME, Muehllehner G (2008). Benefit of time-of-flight in PET: experimental and clinical results. J Nucl Med.

[36] Laffon E, Lamare F, de Clermont H, Burger IA, Marthan R (2014). Variability of average SUV from several hottest voxels is lower than that of SUVmax and SUVpeak. Eur Radiol.

[37] de Pierro AR, Beleza Yamagishi ME (2001). Fast EM-like methods for maximum “a posteriori” estimates in emission tomography. IEEE Trans Med Imaging.

[38] Asma E, Ahn S, Qian H, Gopalakrishnan G, Thielemans K, Ross SG (2012). Quantitatively accurate image reconstruction for clinical whole-body PET imaging.

[39] Ma H, Asma E, Ahn S, Ross S, Manjeshwar R, Wilson D (2013). Clinical evaluation of penalized likelihood reconstruction in whole-body PET studies.

[40] Passalaqua S, Kappadath S, Branch D, Ross S, Stearns C, Schomer D (2014). Qualitative and quantitative evaluation of regularized PET image reconstruction. J Nucl Med.

[41] Boellaard R, Krak NC, Hoekstra OS, Lammertsma AA (2004). Effects of noise, image resolution, and ROI definition on the accuracy of standard uptake values: a simulation study. J Nucl Med.

[42] Burger IA, Huser DM, Burger C, von Schulthess GK, Buck A (2012). Repeatability of FDG quantification in tumor imaging: averaged SUVs are superior to SUVmax. Nucl Med Biol.

[43] Afshar-Oromieh A, Avtzi E, Giesel FL, Holland-Letz T, Linhart HG, Eder M (2015). The diagnostic value of PET/CT imaging with the 68Ga-labelled PSMA ligand HBED-CC in the diagnosis of recurrent prostate cancer. Eur J Nucl Med Mol Imaging.

[44] Heusser T, Mann P, Rank CM, Schafer M, Dimitrakopoulou-Strauss A, Schlemmer HP (2017). Investigation of the halo-artifact in 68Ga-PSMA-11-PET/MRI. PLoS One.

[45] Flavell R, Deller T, Lake S, Carroll P, Hope T, Lawhn Heath C (2017). Scatter artifact with 68Ga PSMA-PET: severity reduced with furosemide diuresis and improved time-of-flight scatter correction. J Nucl Med.

[46] Wangerin KA, Baratto L, Khalighi MM, Hope TA, Gulaka PK, Deller TW, et al. Clinical evaluation of (68)Ga-PSMA-II and (68)Ga-RM2 PET images reconstructed with an improved scatter correction algorithm. AJR Am J Roentgenol. 2018:1–6. 10.2214/AJR.17.19356. [Epub ahead of print]. 10.2214/AJR.17.19356 29873506

[47] Pizzuto DA, Muller J, Muhlematter U, Rupp NJ, Topfer A, Mortezavi A, et al. The central zone has increased (68)Ga-PSMA-11 uptake: “Mickey Mouse ears” can be hot on (68)Ga-PSMA-11 PET. Eur J Nucl Med Mol Imaging. 2018;45(8):1335-43. 10.1007/s00259-018-3979-2. Epub 2018 Mar 9. 10.1007/s00259-018-3979-2 29523924

[48] Puri T, Greenhalgh TA, Wilson JM, Franklin J, Wang LM, Strauss V (2017). [18F]Fluoromisonidazole PET in rectal cancer. EJNMMI Res.

[49] Silva-Rodriguez J, Tsoumpas C, Dominguez-Prado I, Pardo-Montero J, Ruibal A, Aguiar P (2016). Impact and correction of the bladder uptake on 18 F-FCH PET quantification: a simulation study using the XCAT2 phantom. Phys Med Biol.

[50] Mehranian A, Zaidi H (2015). Impact of time-of-flight PET on quantification errors in MR imaging-based attenuation correction. J Nucl Med.

[51] Leynes AP, Yang J, Shanbhag DD, Kaushik SS, Seo Y, Hope TA (2017). Hybrid ZTE/Dixon MR-based attenuation correction for quantitative uptake estimation of pelvic lesions in PET/MRI. Med Phys.

