# Supplementary material for: Quantitative performance and optimal regularization parameter in block sequential regularized expectation maximization reconstructions in clinical 68Ga-PSMA PET/MR
Source: EJNMMI Res. 2018 Jul 27;8:70. doi: 10.1186/s13550-018-0414-4 (PMC6063806; doi:10.1186/s13550-018-0414-4)
Supplement: Supplementary file 1 — Figure S1. Box and whisker plots showing tumor-to-background ratio distributions obtained from lesions in 25 patients. The top panel (a) shows the group with small size and low-uptake lesions (N = 8), the middle panel (b) shows the group with medium size and medium uptake lesions (N = 15), the lower panel (c) shows the group with large size or high-uptake lesions (N = 17). Each subplot shows from left to right the results of the TOF OSEM reconstructions with 28 subsets and 2 and 3 iterations, followed by TOF BSREM reconstructions with regularization parameter β = 150, 200, 250, 300, 350, 400, 450, 500, 550, 600, 700, 800, 900, 1000, and 1200. The tumor-to-background is defined as the SUVmax of the lesion devided by the SUVmean of the background. TOF = time-of-flight, SUV = standardized uptake value, SUVmax = the average of the hottest 5 voxels in the lesion, SUVmean = the average value of the voxels in the background ROI, OSEM = ordered subset expectation maximization, and BSREM = block sequential regularized expectation maximization. (DOCX 174 kb) [file 13550_2018_414_MOESM1_ESM.docx]

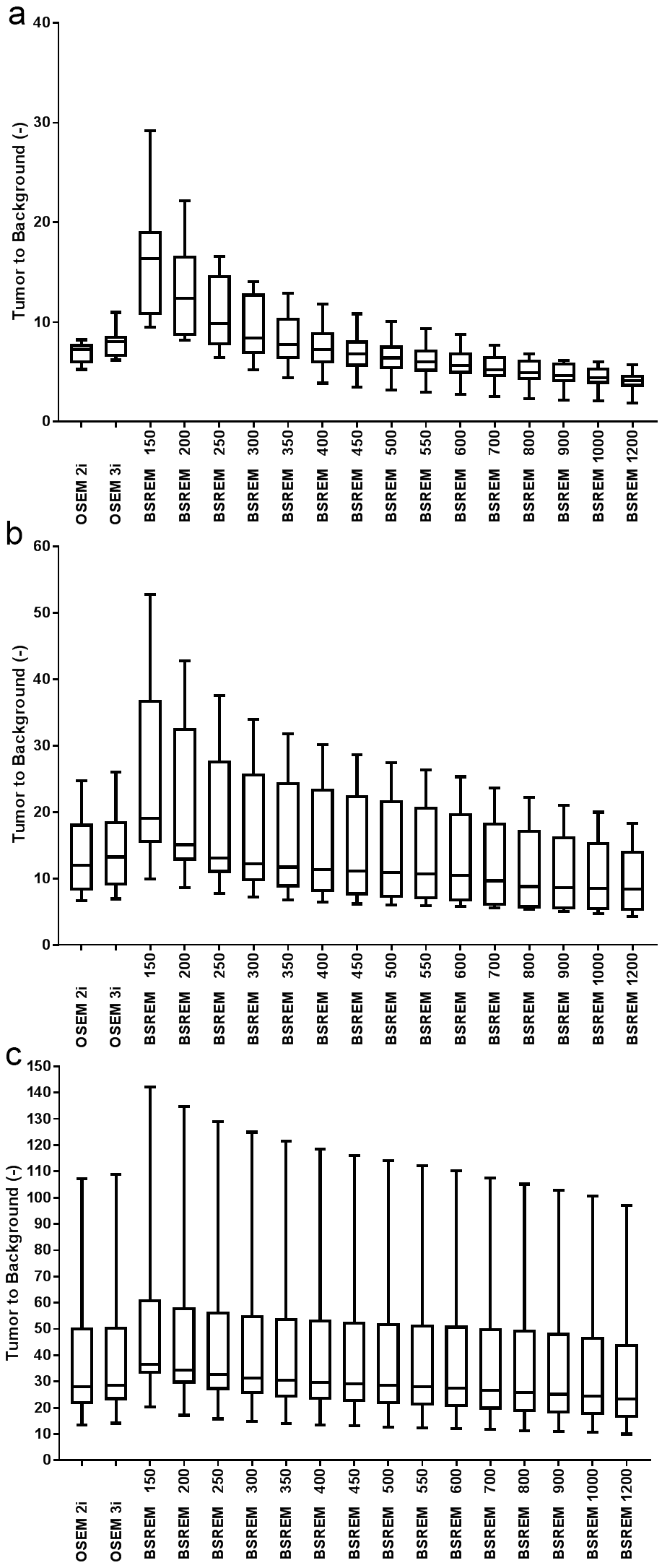


Figure S1: Box and whiskers plots showing tumor-to-background ratio distributions obtained from lesions in 25 patients. The top panel (a) shows the group with small size and low uptake lesions (N=8), the middle panel (b) shows the group with medium size and medium uptake lesions (N=15), the lower panel (c) shows the group with large size or high uptake lesions (N=17). Each subplot shows from left to right the results of the: TOF OSEM reconstructions with 28 subsets and 2 and 3 iterations, followed by TOF BSREM reconstructions with regularization parameter *β* = 150, 200, 250, 300, 350, 400, 450, 500, 550, 600, 700, 800, 900, 1000, 1200. The tumor-to-background is defined as the SUVmax of the lesion devided by the SUVmean of the background.

TOF = time of flight, SUV = standardized uptake value, SUVmax = the average of the hottest 5 voxels in the lesion, SUVmean = the average value of the voxels in the background ROI, OSEM = ordered subset expectation maximization, BSREM = block sequential regularized expectation maximization.
